# Supplementary figures and images for: Neurocysticercosis in Ecuador: Spatial clustering, social determinants, and epidemiological trends (2017–2023)
Source: PLoS Negl Trop Dis. 2025 Jul 8;19(7):e0012205. doi: 10.1371/journal.pntd.0012205 (PMC12251105; doi:10.1371/journal.pntd.0012205)

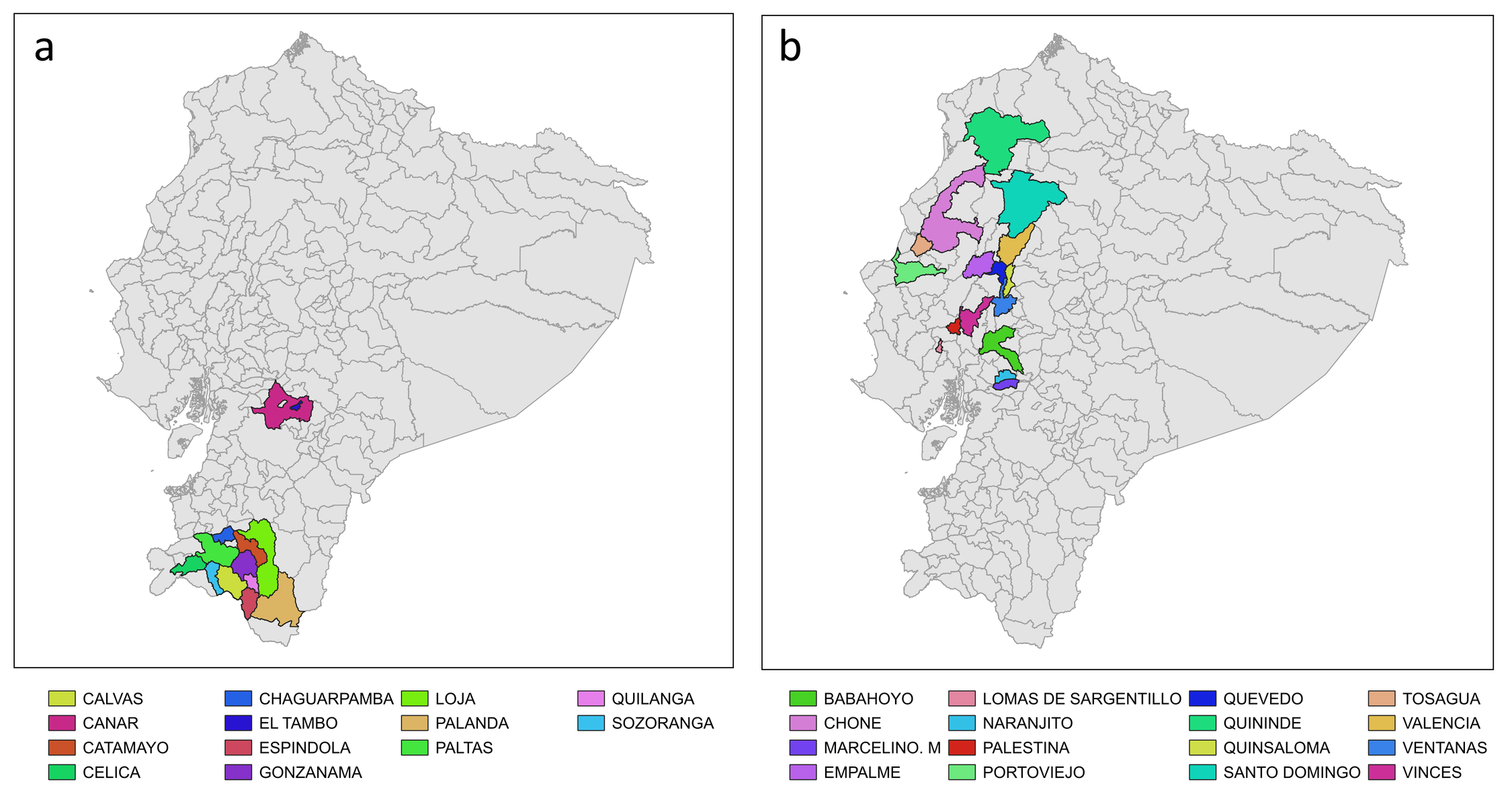

Supplement: S1 Fig — Panel a shows the geographical distribution of the municipalities belonging to hot spots. Panel b shows the geographical distribution of the municipalities belonging to cold spots. Base map layers were sourced from geoBoundaries (ADM-2 shapefile), licensed under CC-BY 4.0 (https://www.geoboundaries.org/countryDownloads.html). (TIF) [file pntd.0012205.s001.tif]

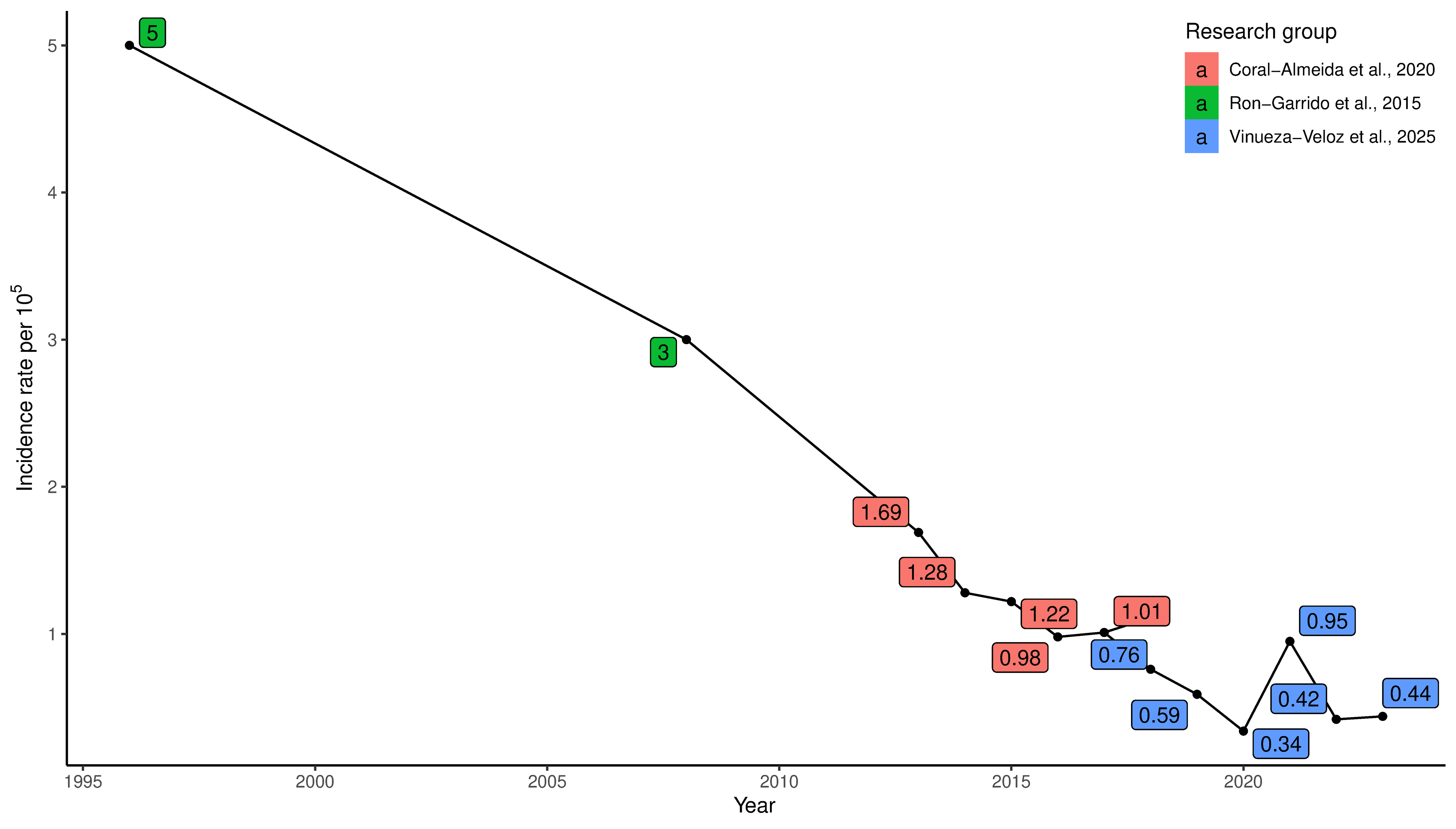

Supplement: S2 Fig — The figure shows the calculated incidences reported by our team and two other research groups in Ecuador (6,8, this study). The displayed incidences are based on hospitalized cases, with the only exception being the year 2021, which includes both outpatient and hospitalized cases (see Methods section). (TIF) [file pntd.0012205.s002.tif]
