# Supplementary material for: Neurocysticercosis in Ecuador: Spatial clustering, social determinants, and epidemiological trends (2017–2023)
Source: PLoS Negl Trop Dis. 2025 Jul 8;19(7):e0012205. doi: 10.1371/journal.pntd.0012205 (PMC12251105; doi:10.1371/journal.pntd.0012205)
Supplement: Attachment — Submitted filename: letter_response_01042025.docx [file pntd.0012205.s004.docx]

**Editor**

***-The reviewers raised concerns about the study's contribution to the existing literature, the lack of acknowledgment of previous similar studies in the introduction, the data sources, figure representations, and the clarity of the English language.***

Thanks for the comment. We have carefully revised the whole text and edited it according to the reviewers’ suggestions.

**Reviewer #1**

***-This manuscript describes the (spatial)incidence of hospitalised NCC cases in Ecuador between 2017-2022. Additionally, associations are investigated with a number of determinants. However, the latter are rather broad, including multiple factors in one variable (e.g. the poverty by unsatisfied basic needs (PUBN) factor includes adequate housing, availability of electricity, water, sanitation; of some factors which do not necessarily impact on T. solium disease transmission, which render the determinant less informative.***

Thank you for the comment. In the original version we tried to use a general indicator of poverty such as PUBN; however, we agree with the reviewer in that other indicators of social exposures might be more informative. In the new version of the paper we instead used more informative indicators (please refer to Table 1) and explored their association with NCC incidence.

***-A lot of information is available on risk factors, and probably data on water and sanitation, free roaming pigs etc would have been much more relevant.***

Thank you for the comment. We agree with the reviewer. We have analyzed the association between drinking water access, sewerage coverage, pig density, and creole pig density in the new version of the paper (please refer to Table 1).

***-On the level of the determinants, this study brings no new information. It is also not clear why the authors solely focus on hospitalized NCC cases, while from the discussion it becomes clear that outpatient data are available as well (and indicate a much higher number of cases).***

Thank you for the comment. We agree with the reviewer in that the previous version of our paper missed quite important information. The contribution of the present study is the throughput identification of spatial clusters of high incidence rate and the exploration of social factors at these clusters as potential drivers of disease transmission. We have tried to highlight this idea in the new version of the manuscript (line 109).

***However, my main concern with this manuscript is whether it is of added value to the current body of knowledge on NCC in Ecuador. A lot of information is already available. For example, the burden and spatial distribution of NCC in Ecuador has been described for 2013-2017 (Coral-Almeida et al. 2020). Also Rodriguez-Rivas et al. studied the epidemiological situation in Ecuador (1998-2019) including associations with the human development index. The studied period of the latter paper mostly overlaps with the current manuscript (2017-2022), and consults the same database (INEC). The currently published knowledge is completely neglected in the introduction. The latter is very general and a clear rationale for the study is missing.***

Thank you for the comment. We agree with the reviewer in that the previous version of our paper missed quite important information. We have cited both papers in the introduction (references 5 and 7; reference 8, line 248 - 251). Adding to the contribution of Coral-Almeida et al. and Rodrígues-Rivas et al., we extended the period of study to 2023. We also compared the distribution of social determinants between clusters of high and low incidence rates. Accordingly, we have rewritten the introduction highlighting what the present study contributes to what is already known (line 99 to 117).

-***The authors collected data on the number of pigs, but not on the farming systems? Pigs kept in enclosed, controlled housing systems represent a totally different/no risk for transmission. In the discussion the authors shortly touch upon backyard farming systems. If data is available (and probably it is from previous studies), I would strongly advice to include those in the analyses.***

Thank you for the comment. Unfortunately, we did not have access to data related to farming systems. However, we included information on population density of creole pigs, which can indirectly inform on farming systems (please refer to Table 1). We found that hot spots for NCC have higher density of creole pigs. Moreover, there was inequality in the distribution of wealth in these clusters, which suggests that the dynamics of disease transmission is complex and does not only depend on farming systems.

***-It is not clear whether specific data on access to safe water, presence and use of latrines were collected or only the more broadly covering data PUBN? Why was this not separately collected, that type of data would have been more informative?***
Thank you for the comment. We agree with the reviewer in that information on access to safe water and sewerage coverage is highly informative. We have included this information in the new version of the manuscript (please refer to Table 1). We would like to clarify that we did not collect data, but instead used already collected data.

***From figure 1 it appears that PUBN and HDI were not collected/available from the years from which NCC data were gathered. Can the authors assume that this has no impact on their study? Why did the authors not include NCC data from the years from which they have PUBN and HDI? This is only shortly mentioned in the discussion.***
Thank you for the comment. We agree with the reviewer in that using data from different years might be dangerous in case of trying to establish causal associations. However, the purpose of the present study is not to establish causal associations between the social determinants and the incidence of NCC, but rather to explore their distribution within the clusters of high and low incidence. To do that we chose to analyze social determinants from the latest data available before 2023 (please refer to Table 1).

***-The correctness of the used databases should be discussed, and the impact this may have on the results. Here NCC incidence is based on hospitalized cases, while many NCC patients would not have been hospitalized, so here we are looking at the tip of the iceberg? Lines 234-241: here the authors highlight differences in results between their study and data from the MPH based on outpatient consultations: it would be good to not only provide the information, but also reflect on what it means. Or: should the authors include these outpatient data in their study for a better representation of NCC in Ecuador?***

Thank you for the comment. We agree with the reviewer. We are aware of the limitations of using inpatient registries, which might imply under-reporting. Unfortunately, we did not have access to outpatients’ registries other than that of 2021. We provide an insight about the limitation of relying mostly on inpatient data in the new version of the discussion section (line 420).

***-More urban than rural hospitalised cases: this is a bit unexpected, and warrants more attention: is there a difference in health seeking behaviour rural versus urban?***

Thank you for the comment. In this new version we have decided to exclude urban or rural origin from the description of cases. This is because we consider that in Ecuador areas considered as urban present typical rural conditions such as lack of access to basic services. Therefore, we base the discussion on more informative variables such as sewerage coverage and economic inequality (Table 1).

***Lines 283-292: the authors are advised to revise this paragraph using knowledge specifically for Taenia spp. There is a number of publications describing influence of environmental factors, including climatic, on the survival rates of Taenia spp.***

Thank you for the comment. We agree with the suggestion. In the current version we have expanded the climate discussion, in this version we discuss the possible role of wastewater use in farming activities in dry climates (such as hot spots) and how this may contribute to the spread of the disease (line 441).

***-The manuscript is written very sloppy, with many mistakes, many abbreviations which makes the manuscript hard to read.***
Thank you for the comment. We have carefully reviewed the whole manuscript and improved readiness.

***-Smaller corrections: (non-exhaustive list, the papers needs a total revision)
 the larva u oncosfera; secondary hosts: (accidental) intermediate host
 cysticerco: cysticercus; references check, eg line 39: (1, del bruto);
General English: eg In addition, NCC is with 1.2 million disability-adjusted life years loss due to its close association with epilepsy (2) with the more one third of the cases in low- and middle income countries; The use of many abbreviations complicates the reading; Graph 1, figure 1: this is confusing; Data source: our world in data, what is the reliability of this source?; In the reference the correct citation should be used, as presented on the website; Line 228: An in our study??; And many other small typos, eg line 150 sount america…, line 258: during xxx to xxx???***

Thank you for the comment. We have carefully reviewed the whole manuscript and improved readiness.

**Reviewer #2**

***-I noticed that your study integrates multiple datasets from various sources, each covering different time periods. For example, the hospital discharge records span a five-year period from 2017 to 2022, while other datasets, such as the poverty indicators, are derived from specific years, like the 2010 census. While this method is understood within the field and offers a comprehensive analysis of neurocysticercosis (NCC) incidence and its socio-economic and environmental determinants, it may be beneficial to explicitly clarify this approach for readers who might not be familiar with these nuances.***

Thank you for the comment. We agree with the reviewer in that integrating multiple datasets and sources might create confusion. We better explain the rationale of the study in the new version of the Methodology Section of the manuscript (line 118).

***A brief explanation regarding the variation in time frames could help in addressing any potential complexities in interpreting the results, and assure that the analysis accurately reflects the relationships between these variables over time***

Thank you for the comment. We agree with the reviewer in that integrating multiple datasets and sources might create confusion. In the new version of the manuscript we clearly state the years for which we have available data on NCC cases (please refer to Table 1).

***-The analysis presented in the results section aligns with the study's analysis plan. The results are clearly and comprehensively presented, with appropriate use of statistical measures to support the findings. However, some figures, particularly Figure 2 and Graph 2, could be enhanced for better clarity and resolution. The tables are well-organized and effectively communicate the study's key findings. The spatial distribution of NCC incidence and the identification of high and low incidence clusters are particularly well-executed, adding significant value to the analysis.***

Thank you for the comment. We have improved the resolution of figures and readiness of tables in the new version of the manuscript.

***The conclusions are generally supported by the data presented. The manuscript successfully discusses the socio-environmental factors influencing NCC incidence and highlights the public health implications of these findings. The limitations of the analysis are mentioned, particularly the use of older poverty data and reliance on hospitalized cases, but these could be expanded upon to provide a more nuanced understanding of the study's constraints. The discussion of how these data can advance our understanding of NCC, especially in the context of low-income countries, is well-handled, though a more explicit focus on public health relevance could strengthen the conclusions.***

Thank you for the comment. We have edited the Limitation and Strengths Section as well as the Conclusion Section accordingly (line 420 to 437).

***-The manuscript could benefit from several editorial modifications to enhance clarity. Consistent labeling of figures (e.g., ensuring "Figure 1" is used instead of "Graph 1") is necessary. The resolution of Graph 2 should be improved to match the quality of other figures. Consider revising the title of Figure 1 to explicitly state the "Incidence Rate of Neurocysticercosis in Cantons of Ecuador." Additionally, the authors might consider replacing the photographs in Figure 3 with an infographic that illustrates the life cycle of the Taenia solium parasite, which would provide more educational value and align better with the manuscript's objectives.***

Thank you for the comment. We have carefully reviewed the whole manuscript and improved readiness of tables and labeling of figures. We have decided to summarize the cycle of the parasite in the introduction (line 87). We decided not including the photographs .

***-Overall, this manuscript makes a significant contribution to the understanding of neurocysticercosis in Ecuador. The study's strengths lie in its comprehensive analysis of socio-economic and environmental factors and its robust methodological approach. However, there are areas for improvement, particularly in the presentation of figures and the discussion of limitations. The manuscript’s novelty lies in its detailed examination of NCC clusters in Ecuador, and its significance is underscored by the potential public health applications of the findings. I recommend a major revision to address the identified concerns, particularly regarding the clarity of data presentation and the expansion of the discussion on study limitations.***

Thank you for the comment. We have carefully reviewed the whole manuscript and improved readiness of tables and labeling of figures. We have also expanded the Discussion Section of the manuscript and study limitations.

**Reviewer #3**

***-Well studied and nicely presented. The objective of the study is clearly stated. The study design is appropriate to address the stated objectives. The population clearly described and appropriate for the concerned study. Statistical analysis used to support conclusions. There is NO concern about ethical or regulatory requirements.***

Thank you for the comment.

***-Figures and tables are appropriate. Figure 3 : should be labelled as A & B for the two pictures. Graphic 1 & 2 should be labelled as Figures and cited appropriately.***
Thank you for the comment. We have carefully reviewed the whole manuscript and improved readiness of tables and labeling of figures.

***-Limitations are identified. Suggesting to add a clear conclusion statement. The public health relevance addressed.***
Thank you for the comment. We have edited Conclusion Section accordingly (line 437).
