# Supplementary material for: Neurocysticercosis in Ecuador: Spatial clustering, social determinants, and epidemiological trends (2017–2023)
Source: PLoS Negl Trop Dis. 2025 Jul 8;19(7):e0012205. doi: 10.1371/journal.pntd.0012205 (PMC12251105; doi:10.1371/journal.pntd.0012205)
Supplement: Attachment — Submitted filename: letter_response_20052025.docx [file pntd.0012205.s005.docx]

**Reviewer 2**

- **The objectives of the study are clearly stated, focusing on the spatial distribution of neurocysticercosis (NCC) incidence in Ecuador and its association with socio-environmental determinants. However, the connection between the hypothesis and the methodology, especially regarding the analysis of "hot spots" and "cold spots," could be further clarified to strengthen the link.**

Response:

Thank you for your observation. We have expanded the rationale in the Introduction to explicitly link the study’s objectives to the methodology. As detailed in the revised manuscript, social determinants of health (SDH) intersect with ecological, economic, and cultural systems to shape disease risk (line 111). By analyzing spatial contrasts between high- and low-incidence clusters, this study aims to uncover how structural inequities create layered vulnerabilities that perpetuate disease persistence. This clarification strengthens the connection between our hypothesis and the use of spatial clustering analysis.

- **The study design is appropriate, utilizing spatial analysis (LISA) and statistical evaluation. However, as it heavily relies on hospital discharge data, the authors should address the potential impact of excluding outpatient data, as it could affect the comprehensiveness of the conclusions.**

Response:

We acknowledge this concern. As noted in the revised version of the manuscript, due to methodological constraints we advocate to interprete incidence rates cautiously (line 362).

- **The population, including hospitalized NCC cases from 2017 to 2023, is clearly defined. The focus on high-incidence clusters is relevant, but a more detailed description of the criteria used for area selection and exclusions would provide additional clarity.**

Response:

Thank you for highlighting this. We explain with more detail how we identified high incidence rate clusters (hot spots) in the Methods section of the new version of the manuscript (line 229).

- **The manuscript does not discuss sample size or power analysis directly, but given the large dataset covering several years and regions, the sample size is likely adequate. It would be beneficial to include a mention of this for transparency and rigor.**

Response:

As this is a nationwide study encompassing all diagnosed NCC cases within Ecuador’s public health system (2017–2023), the sample size inherently represents the full population of interest.

- **The statistical methods, including the use of LISA and regression models, are appropriate. However, the authors should clarify the criteria used to identify "hot spots" and "cold spots" and justify these decisions.**

Response:

The operational definitions for spatial clustering (e.g., significance thresholds, neighborhood criteria) under Anselin’s Local Moran’s I (LISA) methodology are detailed in Methods section of the manuscript (line 229). These parameters align with standard practices in spatial analysis.

- **There are no apparent concerns regarding ethical or regulatory requirements, as the manuscript uses publicly available data. However, it would be helpful for the authors to explicitly mention whether ethical approval was obtained or that ethical considerations were reviewed.**

Response:

This study analyzed anonymized, publicly available secondary data from government repositories. While no specific ethical approval was required for this secondary data analysis, the research was conducted as part of the research project "PROYECTO GUAGUA" (Approval Code: IDIPI-257). This approval code has been added to the Ethics Statement section of the manuscript (line 251).

- **Does the analysis presented match the analysis plan? Yes, the analysis presented largely matches the outlined plan. The focus on spatial clusters and the investigation of social determinants aligns with the initial objectives. However, as previously mentioned, the exclusion of outpatient data warrants further explanation and may need to be addressed more explicitly in the results.**

Response:

Thank you for this observation. We confirm that outpatient cases were not excluded from the analysis. These cases were incorporated using the only nationally available outpatient database. Unfortunately, we have not been able to access to outpatient data from other years.

- **Are the results clearly and completely presented? The results are generally clear, but there is room for improvement in presenting the findings on the spatial distribution of NCC. The use of clearer labeling and more detailed descriptions of figures, particularly related to the spatial clusters, would improve the clarity of the presentation.**
- **Are the figures (Tables, Images) of sufficient quality for clarity? While the figures and tables are mostly clear, there are concerns with the resolution of some figures (e.g., Figure 2 and Graph 2). Improving the resolution and providing more detailed captions would enhance their clarity and make them more accessible to readers.**

Response:

All figures and tables have been revised to meet the journal’s formatting requirements. High-resolution .eps or .tiff files are included.

- **Are the conclusions supported by the data presented? The conclusions are largely supported by the data presented. The identification of spatial clusters and the exploration of socio-environmental factors are valid. However, I recommend strengthening the discussion around the implications of the limitations (e.g., reliance on inpatient data) for the overall conclusions.**

Response:

Berkson’s bias is addressed in multiple sections of the manuscript (line 364, line 373). To partially mitigate this bias (though not eliminate it), outpatient cases were included (line 373). We also clarify that this bias is common to all prior studies, underscoring that clinical data likely represent only the "tip of the iceberg" of the true public health burden (line 362). Furthermore, we explicitly identify this bias as a key limitation and recommend a multi-level validation (line 487).

- **Are the limitations of analysis clearly described? The limitations are mentioned but could be expanded upon. Specifically, the reliance on inpatient data and the potential impact of using different years for poverty indicators and NCC data should be more thoroughly discussed to provide a more nuanced understanding of the study’s constraints.**

Response:

The Limitations and Strengths section indicates that temporal mismatches between SDH indicators and NCC data may hinder the interpretation of the findings (line 478). Moreover, this study does not aim to establish causality; rather, it focuses on identifying contextualized local conditions that perpetuate parasitic transmission (line 119).

- **Do the authors discuss how these data can be helpful to advance our understanding of the topic under study? Yes, the authors highlight how their findings contribute to understanding the spatial epidemiology of NCC in Ecuador. However, they could strengthen the discussion on how these findings might inform public health interventions, particularly in areas with high pig density or inadequate sanitation.**
- **Is public health relevance addressed? The public health relevance is addressed, particularly in the context of disease control and prevention strategies for NCC in Ecuador. However, a clearer discussion of how these findings can inform policy or intervention strategies would further emphasize the importance of the study.**

Response:

We appreciate this suggestion. The revised manuscript now explicitly links findings to actionable interventions, such improving sanitary control of Creole pork as porcine immunization (line 463), gender-focused public health strategies (line 388), and resource redistribution to address inequities (line 466).
