# Supplementary material for: Neurocysticercosis in Ecuador: Spatial clustering, social determinants, and epidemiological trends (2017–2023)
Source: PLoS Negl Trop Dis. 2025 Jul 8;19(7):e0012205. doi: 10.1371/journal.pntd.0012205 (PMC12251105; doi:10.1371/journal.pntd.0012205)
Supplement: Attachment — Submitted filename: letter_response_05062025.docx [file pntd.0012205.s006.docx]

**Editor**

**While revising your submission, please upload your figure files to the Preflight Analysis and Conversion Engine (PACE) digital diagnostic tool, https://pacev2.apexcovantage.com/. PACE helps ensure that figures meet PLOS requirements. To use PACE, you must first register as a user. Registration is free. Then, login and navigate to the UPLOAD tab, where you will find detailed instructions on how to use the tool. If you encounter any issues or have any questions when using PACE, please email PLOS at figures@plos.org. Please note that Supporting Information files do not need this step. If there are other versions of figure files still present in your submission file inventory at resubmission, please replace them with the PACE-processed versions.**

Thanks. We have done that.

**To enhance the reproducibility of your results, we recommend that authors of applicable studies deposit laboratory protocols in protocols.io, where a protocol can be assigned its own identifier (DOI) such that it can be cited independently in the future. Additionally, PLOS ONE offers an option to publish peer-reviewed clinical study protocols. Read more information on sharing protocols at** [**https://plos.org/protocols?utm_medium=editorial-email&utm_source=authorletters&utm_campaign=protocols**](https://plos.org/protocols?utm_medium=editorial-email&utm_source=authorletters&utm_campaign=protocols)

No applicable.

**Reviewer 2**

**Are the objectives of the study clearly articulated with a clear testable hypothesis stated?
Yes. The authors present a clearly articulated objective: to estimate the burden of neurocysticercosis (NCC) in Ecuador using national databases. The hypothesis is implicit but testable—that NCC imposes a measurable health burden, particularly in rural areas, based on hospitalization and outpatient consultation data.**

Thanks for the comment. No change has been done.

**Is the study design appropriate to address the stated objectives?
Partially. The use of national databases is appropriate for an ecological burden estimation study. However, there is a significant issue regarding the Consulta externa dataset, which is only publicly available for 2021. This limitation is not clearly stated in the Methods, which may impact the robustness of the outpatient-related findings. Clarifying the temporal scope of this dataset is essential for transparency and replicability.**

Thanks for the comment. We clearly state this limitation in the Methods section (line 131). We also clearly state that outpatient data was only available for year 2021 (line 145, line 196).

“To enhance case detection we also included outpatient registries, though these were only available for 2021.”

“While *Camas y egresos hospitalarios* was available from 2017 to 2021, *Consulta externa* was only available for 2021.”

“For 2021, both outpatient and hospitalized cases were combined in the numerator, with the denominator corresponding to the municipal population for that year. Outpatient data was unavailable for other years; thus, numerators corresponded only to cases identified in *Camas y egresos hospitalarios* (see Methods section)”

**Is the population clearly described and appropriate for the hypothesis being tested?
Yes. The study includes all registered NCC cases in Ecuador as reported in national databases. The use of population-level data is justified given the ecological nature of the study.**

Thanks for the comment. No change has been done.

**Is the sample size sufficient to ensure adequate power to address the hypothesis being tested?**

**Yes. The study analyzes all NCC cases recorded in nationwide datasets over several years (for hospitalizations) and at least one year (for outpatient consultations). This ensures sufficient data volume, though clarification on the temporal mismatch is needed.**

Thanks for the comment. We clearly state that outpatient data was only available for year 2021 (line 145, line 196).

“While *Camas y egresos hospitalarios* was available from 2017 to 2021, *Consulta externa* was only available for 2021.”

“For 2021, both outpatient and hospitalized cases were combined in the numerator, with the denominator corresponding to the municipal population for that year. Outpatient data was unavailable for other years; thus, numerators corresponded only to cases identified in *Camas y egresos hospitalarios* (see Methods section)”

**Were correct statistical analyses used to support conclusions?**

**Yes. The authors use descriptive and spatial analyses that are standard and appropriate for this type of epidemiological study. However, conclusions drawn from outpatient consultation data must be carefully limited to the available year unless clarified otherwise.**

Thanks for the comment. Indeed, the unavailability of outpatient data for the the other years may hinder case detection, underestimating the burden of NCC. We try to explain better this limitation and its implications to the interpretation of our results in the Discussion section of the new version of the manuscript (line 363, line 374, line 485).

“Epidemiological studies on NCC in Ecuador, including the current investigation, significantly underestimate disease burden due to reliance on hospital-based case identification) (6,8,9,27). ”

“The inclusion of outpatient data for year 2021 offers a closer approximation of the true disease burden and partially mitigates Berkson’s bias for that specific year (27). This methodological adjustment led to a transient spike in NCC incidence (0.95 per 100 000 inhabitants; Fig 1). This peak likely reflects the simultaneous inclusion of both outpatient and hospitalized cases and not a true disease resurgence. Importantly, relying solely on hospitalized data captures fewer than half of cases, even within populations with healthcare access.”

“Reported incidence rates likely underestimate true burden, as case identification relied primarily on hospitalization records. The inclusion of 2021 outpatient data revealed substantial underreporting—case counts increased by ~100% that year, creating an artificial peak in temporal trends. This underestimation is exacerbated by limited healthcare access in remote areas and the subclinical nature of many NCC infections. However, double-counting cannot be ruled out given our use of anonymized public health datasets; patients may transition from outpatient to hospital care without unique identifiers.”

**Are there concerns about ethical or regulatory requirements being met?**

**No. The study uses anonymized, publicly available secondary data from national databases, and no identifiable patient information is used. Ethical standards appear to have been met.**

Thanks for the comment. No change has been done.

**Does the analysis presented match the analysis plan?**

**Yes, overall, the analysis appears to follow the stated methodological approach, which includes extracting NCC case data from national-level hospitalization and outpatient consultation databases, followed by descriptive and spatial analysis. However, there is a critical point that requires clarification. The authors cite the Consulta externa database as a continuous source of outpatient care data, but this dataset is only publicly available for the year 2021. This discrepancy between the stated data coverage and actual availability should be explicitly acknowledged in the Methods section to ensure transparency and replicability. Failing to clarify this undermines confidence in the consistency of the outpatient analysis across time and may affect interpretation of trends.**

Thanks for the comment. Outpatient data (i.e., *Consulta externa*) was only available for 2021. We clarify this in the new version of the Methods section (line 145, line 196).

“While *Camas y egresos hospitalarios* was available from 2017 to 2021, *Consulta externa* was only available for 2021.”

“For 2021, both outpatient and hospitalized cases were combined in the numerator, with the denominator corresponding to the municipal population for that year. Outpatient data was unavailable for other years; thus, numerators corresponded only to cases identified in *Camas y egresos hospitalarios* (see Methods section)”

**-Are the results clearly and completely presented?**

**Yes, the results are generally well-structured and clearly presented, with case counts, demographic breakdowns, and geographical patterns detailed both in the text and figures. Nevertheless, given the potential limitation of the outpatient data to a single year, conclusions drawn from these data should be carefully qualified or reinterpreted to avoid overstating temporal trends.**

Thank you. The implications of this limitation is now acknowledged in the new version of the Discussion section (line 363, line 374, line 485).

“Epidemiological studies on NCC in Ecuador, including the current investigation, significantly underestimate disease burden due to reliance on hospital-based case identification) (6,8,9,27). ”

“The inclusion of outpatient data for year 2021 offers a closer approximation of the true disease burden and partially mitigates Berkson’s bias for that specific year (27). This methodological adjustment led to a transient spike in NCC incidence (0.95 per 100 000 inhabitants; Fig 1). This peak likely reflects the simultaneous inclusion of both outpatient and hospitalized cases and not a true disease resurgence. Importantly, relying solely on hospitalized data captures fewer than half of cases, even within populations with healthcare access.”

“Reported incidence rates likely underestimate true burden, as case identification relied primarily on hospitalization records. The inclusion of 2021 outpatient data revealed substantial underreporting—case counts increased by ~100% that year, creating an artificial peak in temporal trends. This underestimation is exacerbated by limited healthcare access in remote areas and the subclinical nature of many NCC infections. However, double-counting cannot be ruled out given our use of anonymized public health datasets; patients may transition from outpatient to hospital care without unique identifiers.”

**-Are the figures (Tables, Images) of sufficient quality for clarity?**

**Yes, the figures and tables are of acceptable quality. Maps and tables are legible and appropriately labeled. If the outpatient data are indeed limited to one year, it may be helpful to annotate figures related to outpatient consultations to reflect this and avoid confusion for readers.**

Thank you for the comment. To clarify that outpatient data is only available for 2021 we have edited the legend for Fig 1. accordingly.

“Fig 1. National and regional neurocysticercosis (NCC) incidence rate by year. Top panel (National) shows national incidence rate for females and males together (All) and by sex (Female and Male). Bottom panel (Regional) shows regional incidence rate for each of the geographical regions of Ecuador (Amazon, Coast and Highlands). Incidence rate for year 2021 included cases from hospitalization records and outpatient registries (see Methods).”

**The conclusions are generally supported by the data presented; however, the limitation regarding the outpatient data (available only for 2021) should be clearly stated. While the authors discuss the broader implications of NCC distribution and its potential public health impact, the analysis would benefit from a more explicit reflection on the data constraints and how they might affect interpretation. Public health relevance is addressed appropriately.**

Thank you. The implications of this limitation is now acknowledged in the new version of the Discussion section (line 363, line 374, line 485).

“Epidemiological studies on NCC in Ecuador, including the current investigation, significantly underestimate disease burden due to reliance on hospital-based case identification) (6,8,9,27). ”

“The inclusion of outpatient data for year 2021 offers a closer approximation of the true disease burden and partially mitigates Berkson’s bias for that specific year (27). This methodological adjustment led to a transient spike in NCC incidence (0.95 per 100 000 inhabitants; Fig 1). This peak likely reflects the simultaneous inclusion of both outpatient and hospitalized cases and not a true disease resurgence. Importantly, relying solely on hospitalized data captures fewer than half of cases, even within populations with healthcare access.”

“Reported incidence rates likely underestimate true burden, as case identification relied primarily on hospitalization records. The inclusion of 2021 outpatient data revealed substantial underreporting—case counts increased by ~100% that year, creating an artificial peak in temporal trends. This underestimation is exacerbated by limited healthcare access in remote areas and the subclinical nature of many NCC infections. However, double-counting cannot be ruled out given our use of anonymized public health datasets; patients may transition from outpatient to hospital care without unique identifiers.”

**Please clarify the availability of outpatient data used in the analysis. While the source is cited appropriately (Consulta Externa from INEC), it appears that outpatient data are only available for 2021. Since the study spans multiple years, this limitation should be explicitly acknowledged in the methods and discussion sections to ensure transparency and replicability. Aside from this point, the manuscript is clearly written and the figures and tables are of good quality. Minor editorial revisions are suggested for phrasing and consistency. I would recommend Minor Revision pending clarification of the outpatient dataset clarification of the outpatient dataset.**

Thank you. The implications of this limitation is now acknowledged in the new version of the Methods and Discussion sections (Please refer to the previous comments).

**I appreciate that the authors have addressed my previous comments with clarity and made the necessary adjustments. The manuscript presents a relevant and timely analysis of neurocysticercosis using national data sources in Ecuador, contributing to the understanding of its epidemiology and public health burden. However, a key methodological clarification remains pending. While the sources of inpatient and outpatient data are described, it appears that outpatient data ("Consulta Externa") is only available for the year 2021. This limitation should be explicitly stated in the Methods section to ensure transparency and allow future replication of the study. Once this issue is addressed, I consider the manuscript close to acceptance with minor revisions.**

Thank you. The implications of this limitation is now acknowledged in the new version of the Methods and Discussion sections (Please refer to the previous comments).
